# Selective Deeply Supervised Multi-Scale Attention Network for Brain Tumor Segmentation

**DOI:** 10.3390/s23042346

**Published:** 2023-02-20

**Authors:** Azka Rehman, Muhammad Usman, Abdullah Shahid, Siddique Latif, Junaid Qadir

**Affiliations:** 1 Center for Artificial Intelligence in Medicine and Imaging, HealthHub Co., Ltd., Seoul 06524, Republic of Korea; 2 Department of Computer Science and Engineering, Seoul National University, 1 Gwanak-ro, Gwanak-gu, Seoul 08826, Republic of Korea; 3 Faculty of Health, Engineering and Sciences, University of Southern Queensland, Springfield 4300, Australia; 4 Department of Computer Science and Engineering, College of Engineering, Qatar University, Doha 2713, Qatar

**Keywords:** brain tumor segmentation, 3D segmentation, selective deep supervision

## Abstract

Brain tumors are among the deadliest forms of cancer, characterized by abnormal proliferation of brain cells. While early identification of brain tumors can greatly aid in their therapy, the process of manual segmentation performed by expert doctors, which is often time-consuming, tedious, and prone to human error, can act as a bottleneck in the diagnostic process. This motivates the development of automated algorithms for brain tumor segmentation. However, accurately segmenting the enhanced and core tumor regions is complicated due to high levels of inter- and intra-tumor heterogeneity in terms of texture, morphology, and shape. This study proposes a fully automatic method called the selective deeply supervised multi-scale attention network (SDS-MSA-Net) for segmenting brain tumor regions using a multi-scale attention network with novel selective deep supervision (SDS) mechanisms for training. The method utilizes a 3D input composed of five consecutive slices, in addition to a 2D slice, to maintain sequential information. The proposed multi-scale architecture includes two encoding units to extract meaningful global and local features from the 3D and 2D inputs, respectively. These coarse features are then passed through attention units to filter out redundant information by assigning lower weights. The refined features are fed into a decoder block, which upscales the features at various levels while learning patterns relevant to all tumor regions. The SDS block is introduced to immediately upscale features from intermediate layers of the decoder, with the aim of producing segmentations of the whole, enhanced, and core tumor regions. The proposed framework was evaluated on the BraTS2020 dataset and showed improved performance in brain tumor region segmentation, particularly in the segmentation of the core and enhancing tumor regions, demonstrating the effectiveness of the proposed approach. Our code is publicly available.

## 1. Introduction

Brain tumors, also known as neoplasms of the brain, are caused by the abnormal and uncontrolled growth of neural cells within the cranial cavity. These malignant growths are severe pathological conditions that affect the nervous system. According to the National Brain Tumor Society (NBTS), approximately 87,240 people in the United States are diagnosed with a primary brain tumor each year, including malignant and non-malignant tumors. Additionally, approximately 18,020 people die each year from brain tumors and other nervous system tumors in the U.S. [1]. These figures are on the rise year after year. Gliomas, the most common primary brain tumors in adults, severely damage the central nervous system. Gliomas are typically classified into two categories: low-grade (LGG) and high-grade (HGG), with HGG being more aggressive and spreading rapidly with a life expectancy of two years or less for patients with HGG [2].

**Figure 1:**
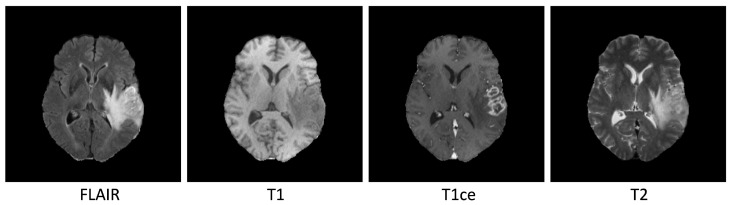
Illustrations of brain tumor regions in an MRI slice from the BraTS 2020 database. From **left** to **right**: FLAIR, T1, T1ce, and T2 slices.

Magnetic resonance imaging (MRI) has greatly improved the visualization of brain tumors. Detailed images of brain tumors can be obtained by using MRI sequences, such as T1-weighted, T2-weighted, T1-weighted with contrast enhancement (T1c), and fluid-attenuated inversion recovery (FLAIR) images, as shown in Figure 1. Automated segmentation of brain tumors is a complex task due to the wide range of variations in the shape, size, and location of tumors among individuals. Additionally, the presence of irregular boundaries between adjacent structures and subtle intensity gradients can make the segmentation process difficult, particularly for core and enhanced tumors. Despite these challenges, it is crucial to strive for precise segmentation of tumors, as it is essential for diagnostic, therapeutic, and surgical purposes [2].

In recent times, many proposals for the automated segmentation of brain tumors have been put forth [3,4,5,6]. The emergence of deep learning (DL)-based techniques has resulted in marked improvements in the performances of a variety of computer vision-related tasks [7,8], particularly in the realm of healthcare-related challenges [9,10,11,12]. The use of deep learning-based methods for brain tumor regions segmentation tasks has notably improved the segmentation of enhanced tumor (ET), whole tumor (WT), and tumor core (TC) regions [13,14]. However, due to their smaller sizes, irregular shapes, and similar textures to the surrounding tissues, enhanced and core tumors remain complex challenges in terms of reliable segmentation [2]. To date, none of the currently available methods, including [2,13,15,16,17,18], have achieved the same level of performance for ET and TC region segmentation (regarding the whole tumor region).

In the realm of DL-based methods, 3D convolutional neural networks (CNNs) are widely used for the demanding task of volumetric segmentation. In comparison, 2D-CNNs demand fewer computational resources and training samples for detecting tumors in individual slices, but their performance in segmentation is limited by their incapacity to effectively process the crucial 3D sequential information required for volumetric segmentation [2]. To take advantage of the strengths of both 2D and 3D architectures, a hybrid method was developed that utilizes significantly less computational power than 3D CNNs but still fully leverages contextual data between slices.

The use of complex architectures for brain tumor segmentation can sometimes lead to issues, such as vanishing gradients and convergence problems. One solution to these issues is called deep supervision, which involves adding auxiliary classifiers to the early layers of the network. However, this approach can actually decrease the model’s performance because the early layers are responsible for extracting low-level features, and the added classifiers disrupt this process [19]. Additionally, simple auxiliary classifiers may not provide enough supervision to improve the model’s performance. To address these limitations, we propose the use of *selective deep supervision* (SDS) for effective brain tumor segmentation. In contrast to deep supervision, selective deep supervision enables our proposed network to gradually increase the complexity of the task and to only supervise the final layers with all tumor regions. Our proposed architecture uses both 2D and 3D MRI scans as inputs and employs two encoding branches to extract information at multiple scales. The global features from the 3D input and local features from the 2D image are combined and attention units are applied to suppress redundant features.

Consequently, the main contributions of this work are enumerated as follows.

- This study proposes a novel selective deeply supervised multi-scale attention network (SDS-MSA-Net) framework that combines global and local features to improve the performance of brain tumor segmentation.
- The proposed model incorporates selective deep supervision as a novel training approach to improve the performance of the model for the task at hand. By adding auxiliary outputs at various levels of the network, we aim to achieve improved performance, faster convergence, and better generalization of the model.
- The presented methodology underwent a comprehensive evaluation for the task of brain tumor segmentation on the BraTS2020 dataset [13]. Our framework demonstrates substantial progress in the segmentation of both the enhanced and core brain tumor regions, as evidenced by the improvement in the Dice score, which serves as a metric for the efficacy of our proposed framework.

The structure of the subsequent paper is as follows: In Section 2, a comprehensive review of relevant literature is presented. The materials and methods utilized in this study are outlined in Section 3. The evaluation of the results, accompanied by an analysis and discussion, are presented in Section 4. The paper culminates with a conclusion in Section 6.

## 2. Related Work

Deep learning-based approaches for brain tumor segmentation have gained significant traction, especially after the Multimodal Brain Tumor Segmentation competition in 2012, which was conducted in collaboration with the Medical Image Computing and Computer-Assisted Intervention (MICCAI) Society. Here, we will provide a quick overview of the newly suggested deep learning-based strategies.

In the 2019 MICCAI Brain Tumor Segmentation competition, the top results were achieved by a UNet-based cascade network proposed by Jiang et al. [20]. This approach utilized two decoders, both of which were nearly identical in their architecture, but employed different techniques, such as trilinear approximation and deconvolution, to perform image reconstruction. Furthermore, the encoder was normalized by incorporating an internal branch within the second phase of the network, resulting in exceptional precision. This exemplifies how the integration of an additional branch within the decoder can reduce the risk of overfitting and enhance the overall performance of the model.

The work by Myronenko et al. [21] proposed the use of an asymmetrical UNet architecture for brain tumor segmentation. This approach involves the use of an extensive encoder for feature extraction and a smaller decoder for label reconstruction. Their methodology proved to be effective on the BraTS 2018 dataset, resulting in them earning first place in the competition. A noteworthy contribution of their work is the incorporation of a variational autoencoder (VAE) variant to regularize the encoder and enhance the model’s generalization capability. Similarly, Isensee et al. [22] showed that even slight modifications to a conventional UNet architecture can significantly improve its effectiveness. They also employed additional training data and kept the feature map sizes as small as possible before sampling from the decoder.

Pereira et al. [3] examined the segmentation of brain tumors in MRI images through the utilization of small 3×3 kernels derived from the VGGNet architecture, in conjunction with intensity normalization and data augmentation techniques. They employed a variety of CNN designs for both high- and low-grade tumors, dividing the tumor into the enhancing and core regions. Their hypothesis was tested on the BraTS 2013 dataset, resulting in a Dice similarity rate of 0.88, 0.83, and 0.77 for the full, core, and enhancing areas, respectively. This approach earned them the top spot in the publicly available BraTS 2013 competition. Similarly, Zhao et al. [23] proposed a method that combines fully convolutional neural networks and conditional random fields to accomplish brain tumor segmentation. This strategy was evaluated on BraTS datasets from 2013, 2015, and 2016, yielding satisfactory performance. However, the training process of this method is computationally demanding.

Mostefa et al. [4] presented a triad of fully automated methods for brain tumor segmentation utilizing the iterative optimization technique. The approach was evaluated on the BraTS-2017 dataset, achieving a commendable overall Dice score of 0.88. Havaei et al. [5] proposed an automated brain tumor segmentation method based on 2D-CNN, comprising two pathways, one global and one local, which employed convolution kernels of varying dimensions to extract diverse contextual feature information. The authors evaluated their method using the BraTS 2013 dataset, which yielded high precision. However, the main drawback of patch-wise designs is the lack of spatial continuity. Dong et al. [6] proposed an automated approach for brain tumor segmentation utilizing a 2D version of the UNet model. They evaluated their proposed model using data from BraTS 2015 and found it to be sufficiently accurate. However, the limited computational capacity of UNet may impede its ability to effectively learn image features.

The attention mechanism, which has recently gained significant attention due to its exceptional performance, is an approach that allows for the targeted identification of critical information while obscuring irrelevant data. This technique has been applied to a wide range of computer vision tasks, such as medical image segmentation [24] and medical classification [25]. Researchers have been exploring ways to incorporate the attention mechanism into deep neural networks with the goal of enhancing the accuracy of image segmentation and classification methods. One example is the work by Wang et al. [26], who used a residual attention network to generate attention-aware features from various inputs and found that as more layers were added, the classification accuracy increased. Similarly, Zhang et al. [24] achieved promising results in ventricle segmentation using a modified fully convolutional network and multi-attention modules.

## 3. Materials and Methods

The proposed selective deeply supervised multi-scale attention network (SDS-MSA-Net)-based framework performs brain tumor segmentation by utilizing two different types of inputs, i.e., 2D and the 3D patches of MRI scans, to leverage 2D contextual information and 3D sequential spatial information. Firstly, we performed pre-processing of the scan to normalize the dimensions and enhance the tumor contrast. Later, we performed slice-by-slice segmentation by using the proposed SDS-MSA-Net architecture. Finally, 2D segmentation results were concatenated to produce the 3D volumetric segmentation of brain tumors. The details of each step and component of the proposed scheme are described in the following subsections.

### 3.1. Data and Preprocessing

In this study, the BraTS dataset is utilized, which comprises multimodal magnetic resonance imaging (MRI) scans, represented in NIfTI file format. The BraTS data encompasses various modalities of MRI, including native (T1), post-contrast T1-weighted (T1Gd), T2-weighted (T2), and fluid-attenuated inversion recovery (FLAIR) images. The scans were acquired using different clinical protocols and various scanners from multiple institutions. The imaging datasets were manually segmented by one to four raters, adhering to a consistent annotation protocol, and the annotations were vetted by experienced neuro-radiologists. The annotations include the GD-enhancing tumor, the peritumoral edema, and the necrotic and non-enhancing tumor core. The BraTS dataset has various versions based on the year of release, with BraTS2020, BraTS2019, and BraTS2017 being the most popular in academic research. The statistical details about each dataset are provided in Table 1. The dimensions of each scan in the BraTS datasets were normalized to 240×240×155.

**Figure 2:**
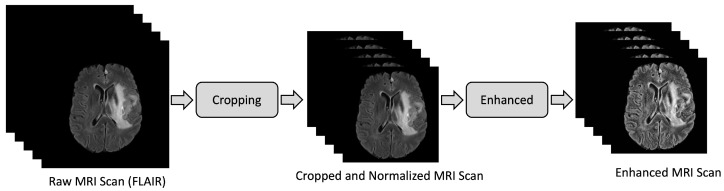
The illustration of the preprocessing stage, which includes scan refinement and image enhancement using cropping and histogram equalization, respectively.

For this study, the BraTS2020 dataset was utilized, as it is the most extensive subset of the BraTS datasets. The task of brain tumor segmentation is known to be particularly challenging owing to the intricate anatomy of the brain, variations in intensity, and the impact of imaging quality. To improve the overall quality of the data, pre-processing techniques were employed as described in Figure 2. Similar to previous studies, we applied histogram equalization to enhance the contrast of the brain tumor. This technique has already been shown to improve the performance of brain tumor segmentation [27].

In this investigation, a pre-processing methodology was utilized to refine and enhance the quality of images (initially provided in a compressed format) obtained from the BraTS dataset. The initial steps involved the removal of blank slices from both ends, followed by the cropping of blank spaces within each slice, yielding a refined image of dimensions 160×160. Subsequently, the histogram equalization (HE) technique was employed to further improve the image quality. This widely used method for contrast enhancement involves the utilization of the cumulative distribution function (CDF) to map the input image’s intensity levels to new intensity levels, thereby effectively expanding the intensity to its full dynamic range. Furthermore, the intensities were normalized between −1 and 1 to further enhance the contrast. To take advantage of the 3D sequential information, adjacent slices were concatenated to generate a three-channel image.

### 3.2. Selective Deeply Supervised Multi-Scale Convolutional Neural Network

**Figure 3:**
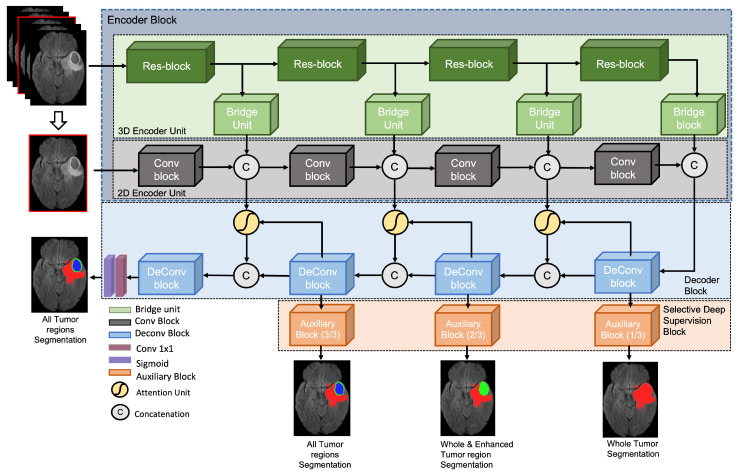
Selective deeply supervised multi-scale attention network (SDS-MSA-Net) takes 2D and 3D inputs to segment three types of brain tumor regions. SDS-MSA-Net produces four outputs, which enable it to be trained with the selective deep supervision technique.

The proposed SDS-MSA-Net (Figure 3) framework utilized two types of inputs, i.e., 2D and 3D patches of brain MRI scan, for which two encoding branches extracted the meaningful information at multi-scale. These multi-scale features were fed to attention units for suppressing the redundant features to feed into the decoder block. The decoder block is responsible for upsampling the input features coming from bottleneck units of encoder blocks as well as the refined features extracted from attention units at various levels. Four different outputs were generated (including three auxiliary outputs from intermediate layers and one from the final layer) to train the model with selective deep supervision. To generate the auxiliary outputs, auxiliary blocks were utilized, which extracted the features from various layers of the decoder block to generate the brain tumor segmentation masks. Each component of the proposed framework is discussed in the following subsections.

#### 3.2.1. Encoder Block

Brain tumors can vary in the shape, size, and texture, making it challenging to accurately segment the tumor regions using only 2D images. To address this issue, a method is proposed that utilizes a 3D encoder to incorporate the sequential information from multiple slices of an MRI scan. The encoder takes a 3D sub-volume consisting of five consecutive slices around the slice for which segmentation is to be performed. The proposed architecture includes two encoder units: a 3D encoder to extract high-level, global features and a 2D encoder to learn low-level, local features that allow the network to focus specifically on the tumor present in the targeted slice.

**Figure 4:**
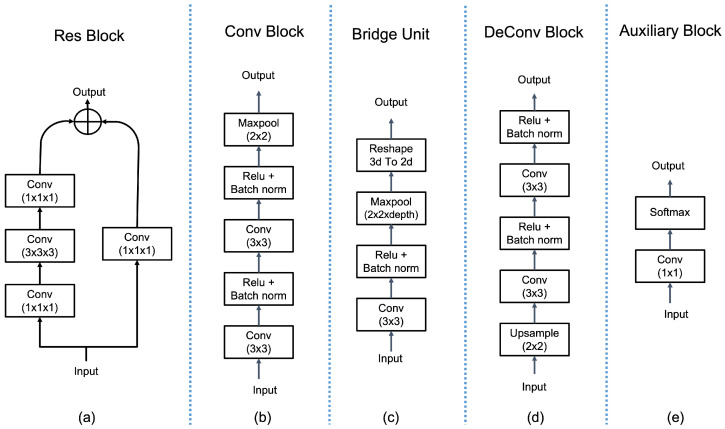
Illustration of the architecture of Res block, Conv block, bridge block, DeConv block, and auxiliary block. (**a**) Res blocks and (**c**) bridge blocks are used in the 3D encoding unit to extract and to downscale the dimensions of the meaningful features, respectively; (**b**) Conv blocks are employed in the 2D encoding unit; (**d**) DeConv block is used in the decoder block to upscale the refined features; finally, (**e**) the auxiliary block employed in the SDS block to immediately upscale the features from intermediate layers of the decoder block to produce the segmentation mask of the selected brain tumor region(s).

The first 3D sub-volume is extracted around nth slice consisting of five slices, i.e., two from forward and backward directions. This 3D patch is fed to a 3D encoder unit that consists of four residual blocks (Res blocks); each block is followed by another Res block and bridge unit. The bridge units connect the 3D encoder unit to the 2D encoder. The architecture of Res block is inspired [28], which consists of Path A and Path B, as shown in Figure 4a. Path A is comprised of three consecutive convolutions, with kernel sizes of 1×1, 3×3, and 1×1, respectively. The first convolution utilizes a stride of 2, reducing the input’s width and height by half. The final convolution features an output channel that is four times larger than the preceding two, forming what is referred to as a bottleneck structure. Path B employs a 1x1 convolution with a stride of two to transform the input’s shape to match that of Path A, thus allowing for the summation of both paths’ outputs to produce the output of the downsampling block. A Res block is similar to the downsampling block, but utilizes only convolutions with a stride of 1. The architecture of the bridge block is depicted in Figure 4c, which includes a convolutional layer followed by a ReLU activation function and a batch normalization layer. The final steps involve the application of max-pooling and a reshape operation, reducing the dimensions of the output features.

To incorporate the local 2D contextual features, the 2D slice is fed to a 2D encoder unit that learns the meaningful features by reducing the 2D dimensions using convolutional blocks (Conv blocks). The 2D encoder unit consists of four Conv blocks that are connected via a concatenation layer that combines the high-level 3D features and low-level 2D features coming from the 3D encoder unit and Conv blocks, respectively. Conv block architecture is shown in Figure 4b which consists of two sets, containing a convolutional layer followed by ReLU and batch normalization layers, and a max-pooling layer.

#### 3.2.2. Decoder Block

**Figure 5:**
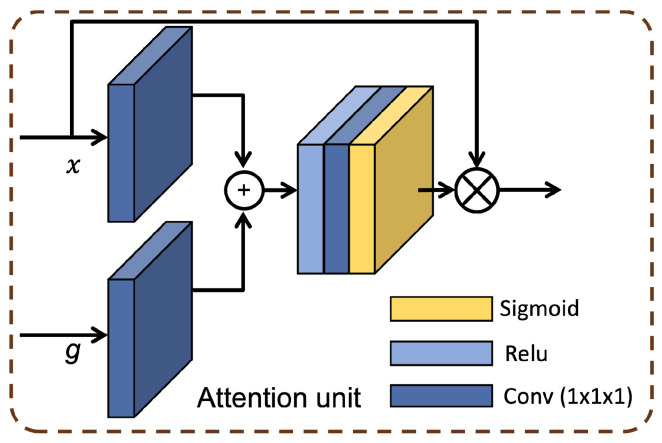
The schematic of the attention unit (AU) that uses additive attention is illustrated. AG is being utilized in the decoder block in the proposed SDS-MSA-Net (Figure 3). The input features (*x*) are scaled with attention coefficients (α) computed in AU. Spatial regions are selected by analyzing both the activations and contextual information provided by the gating signal (*g*), which is collected from a coarser scale. AUs are employed in the proposed MSA-Net at the decoder block to refine the coarse features coming from the encoder block.

The 2D and 3D coarse features extracted by 2D and 3D encoding units are concatenated at four levels and fed to the decoder block. At the decoder block, the attention units are first employed, which filter the redundant features [29]. Attention coefficients, $\alpha_{i}\in \left[0,1\right]$, identify salient image regions and prune feature responses to preserve only the activations relevant to the specific task. The architecture of the attention unit (AU) is demonstrated in Figure 5. The output of AUs is the element-wise multiplication of input feature maps and attention coefficients: ${\hat{x}}_{i,c}^{l}=x_{i,c}^{l}\cdot \alpha_{i}^{l}$. In a default setting, a single scalar attention value is computed for each pixel vector $x_{i}^{l}\in R^{F_{l}}$ where $F_{l}$ corresponds to the number of feature maps in layer *l*. Each AU learns to focus on a subset of target structures. As shown in Figure 5, a gating vector $g_{i}\in R^{F_{g}}$ is used for each pixel *i* to determine focus regions. The gating vector contains contextual information to prune lower-level feature responses. Similar to Oktay et al. [29], additive attention is being utilized to obtain the gating coefficient to achieve higher accuracy than multiplicative attention. Additive attention is formulated as follows:

(1) $$\begin{array}{c} q_{att}^{l}=\psi^{T}\left(\sigma_{1}\left(W_{x}^{T}x_{i}^{l}+W_{g}^{T}g_{i}+b_{g}\right)\right)+b_{\psi }\\ \alpha_{i}^{l}=\sigma_{2}\left(q_{att}^{l}\left(x_{i}^{l},g_{i};\Theta_{att}\right)\right) \end{array}$$

where $\sigma_{2}\left(x_{i,c}\right)=\frac{1}{1+\exp\left(-x_{i,c}\right)}$ correspond to sigmoid activation function. AU is characterized by a set of parameters $\Theta_{att}$ containing: linear transformations $W_{x}\in R^{F_{l}\times F_{\mathrm{int}}},W_{g}\in R^{F_{g}\times F_{\mathrm{int}}}$, $\psi \in R^{F_{\mathrm{int}}\times 1}$ and bias terms $b_{\psi }\in R,b_{g}\in R^{F_{\mathrm{int}\;}}$. The linear transformations are computed using channel-wise 1×1×1 convolutions for the input tensors.

The refined features coming from attention units are concatenated with the outputs of DeConv blocks at three levels. The DeConv block upsamples the features while learning underline patterns associated with brain tumor regions that are crucial for accurate segmentation. The architecture of the DeConv block is demonstrated in Figure 4d; it contains one upsample layer followed by two sets of convolutional layers, ReLU, and batch normalization layer. Each DeConv block is connected with an SDS block, except the deepest DeConv block, which produces the final segmentation mask for whole, enhanced, and core tumor regions.

#### 3.2.3. Selective Deep Supervision Block

To segment the brain tumor from the complicated brain environments in volumetric MRI scans, the proposed multi-scale attention network is designed with relatively more layers to encode highly representative features. However, training such a deeper network is intrinsically a challenging task due to the notorious problem of gradients vanishing which would make the loss back-propagation ineffective and hamper the convergence of the training process [30]. Concretely, it has been observed that back-propagated gradients become smaller as it moves from the deepest layer to the input layer [31]. This can result in varying gradient magnitudes among different layers of the network, leading to issues with optimization and slower training. To overcome this issue, Dou et al. [32] proposed deep supervision as a training scheme in which auxiliary supervision is added at multiple intermediate layers of the network, rather than just at the final output layer. This allows the network to learn more fine-grained features and reduce the risk of overfitting. The additional supervision can be in the form of output layers or loss functions at intermediate layers, which are trained to predict the same target output as the final layer. However, standard deep supervision negates the intuition of CNNs about learning different types of features at various scales to effectively exact the underlying patterns in the given data.

To counter the challenges inherent in conventional deep supervision, an extended version of deep supervision, referred to as SDS, is proposed for brain tumor segmentation. Similar to deep supervision, lower-level and middle-level features from the decoder block are first upscaled using additional deconvolutional layers, known as the auxiliary block, as shown in Figure 4. The hierarchical structure of the brain tumor region, where the core and enhanced tumor regions are subsets of the entire tumor, is utilized to supervise the initial layers of the decoder block through the simpler task of segmenting the entire tumor region.

To tackle the problem of unstable gradient changes during training, the use of explicit supervision for the hidden layers is proposed in a 3D fully convolutional network. This is accomplished by the upscale lower- and middle-level feature volumes through the addition of deconvolutional layers. Then, the softmax function is applied to these full-sized feature volumes to generate dense predictions. These predictions are compared to the ground truth segmentation masks, and their classification errors are calculated as negative log-likelihood. These auxiliary losses, in conjunction with the loss from the final output layer, are used to optimize the back-propagation of gradients for more efficient parameter updates during each iteration.

The layers in the network that have feature volumes directly connected to the final output layer are referred to as the mainstream network. The weights in the *l*-th layer of the mainstream network are represented by $w^{l}$, where *l* ranges from 1 to *L*. The set of weights in the mainstream network is denoted as $W=(w^{1},w^{2},\ldots ,w^{L})$. With $p\left(t_{i}|x_{i};W\right)$ representing the probability prediction of a voxel $x_{i}$ after the softmax function in the last output layer, the negative log-likelihood loss can be formulated as mentioned in the Equation (2).

(2) $$L\left(X;W\right)=\sum_{x_{i}\in X}-\log p\left(t_{i}|x_{i};W\right)$$

where X represents the training database and $t_{i}$ is the target class label corresponding to the voxel $x_{i}\in X$. Here, in contrast to the standard deep supervising, different $t_{i}$ for intermediate layers were used, i.e., $t_{1}$ corresponds to the whole tumor while $t_{2}$ also includes the enhanced tumor region label. Different weights are assigned with respect to the depth while training the network.

In contrast, the layers that generate supplementary dense predictions are referred to as branch networks. The concept of SDS is specifically introduced through these branch networks. To incorporate deep supervision from the *d*-th hidden layer, the weights of the first *d* layers in the decoder block are denoted as $W_{d}=\left(w^{1},w^{2},\ldots ,w^{d}\right)$. The weights that connect the *d*-th layer’s feature volumes to the dense predictions are represented by ${\hat{w}}_{d}$. The auxiliary loss for deep supervision can then be written as:

(3) $$L_{d}\left(X;W_{d},{\hat{w}}_{d}\right)=\sum_{x_{i}\in X}-\log p\left(t_{i}|x_{i};W_{d},{\hat{w}}_{d}\right).$$

Finally, the weights *W* and all ${\hat{w}}_{d}$ are optimized using the back-propagation algorithm by minimizing the overall objective function:

(4) $$L=L\left(X;W\right)+\sum_{d\in D}\eta_{d}L_{d}\left(X;W_{d},{\hat{w}}_{d}\right)+\lambda \left({∥W∥}^{2}+\sum_{d\in D}{∥{\hat{w}}_{d}∥}^{2}\right)$$

where $\eta_{d}$ is the balancing weight of $L_{d}$, which is decayed during learning, and D is the set of indexes of all the hidden layers, which are equipped with deep supervision. The first term corresponds to the output predictions in the last output layer. The second term is from SDS. The third term is the weight decay regularization and λ is the trade-off hyperparameter. In each training iteration, the inputs to the network are large volumetric data, and the error back-propagations from these different loss components are simultaneously conducted.

#### 3.2.4. Implementation Details and Training Strategy

The proposed SDS-MSA-Net was implemented using the TensorFlow framework and the stochastic gradient descent (SGD) was used to minimize the error. The training was carried out on an Nvidia RTX Titan GPU with an input size of 224×224 and a batch size of 8 for 600 epochs. The model was initialized with random weights and trained with a learning rate (lr) of 0.001. To prevent overfitting, early stopping was implemented with the patience set to 10 epochs (the number of training cycles that the model would continue to run even after the performance on the validation set stopped improving). The code of our model is publicly available at https://github.com/Azkarehman/SDS-MSA-Net.git (accessed on 19 February 2023).

### 3.3. Performance Measures

The four evaluation parameters used to assess the performance of the proposed framework are as follows:

- *Dice Similarity Coefficient:* The evaluation of the proposed framework’s performance utilizes the Dice similarity coefficient (DSC) [33]. The DSC measures the degree of overlap between the ground truth mask and the predicted mask, with values ranging from 0 to 1. A value of 1 represents complete overlap and a value of 0 represents no overlap. The DSC is defined as follows:
  (5) $$DSC=\frac{2*Y^{\prime }\cap Y}{Y^{\prime }\;\cup \;Y}$$
  where $Y^{\prime }$ and *Y* are the predicted segmentation mask and reference segment mask, respectively.
- *Sensitivity:* To measure the pixel classification performance proposed framework, the used sensitivity (SEN) can be defined as follows:
  (6) $$SEN=\frac{Y^{\prime }\cap Y}{Y}$$
- *Specificity:* To measure the correctness of the segmentation area produced by the proposed framework, the used Specificity can be defined as follows:
  (7) $$Specificity=\frac{Y^{\prime }\cap Y}{Y^{\prime }}$$
- *Hausdorff Distance:* The Hausdorff Distance (HD) is a widely used metric in the assessment of medical segmentation [34]. The Hausdorff distance is an important measure in brain tumor segmentation because it provides a quantitative way to evaluate the similarity between two sets of points, such as the ground truth segmentation and the predicted segmentation. It calculates the differences between two sets of points, with the directed Hausdorff distance between two sets ($S_{Ref}$ and $Y^{\prime }$) defined as the maximum distance between each point x∈Y and its nearest neighbor $y\in Y^{\prime }$.
  (8) $$H\left(Y,Y^{\prime }\right)=max_{x\in Y}\{min_{y\in Y^{\prime }}\{∥x,y∥\}\},$$
  where ∥x,y∥ is any norm, i.e., the Euclidean distance function. Note that $H\left(Y,Y^{\prime }\right)\neq H\left(Y^{\prime },Y\right)$ and, thus, the directed Hausdorff distance is not symmetric. The Hausdorff distance in both directions is the maximum of the directed Hausdorff distances and, thus, it is symmetric. HD is given by:
  (9) $$HD\left(Y,Y^{\prime }\right)=max\left\{H\left(Y,Y^{\prime }\right),H\left(Y^{\prime },Y\right)\right\}.$$

## 4. Results and Discussion

### 4.1. Benchmarking Results

The performance of the proposed framework, SDS-MSA-Net, was benchmarked against conventional attention-Unet and 3D multi-scale architectures. To assess the contribution of each component, 2D attention UNet, 3D multi-scale network with 2D output, multi-scale attention architecture with a traditional training scheme, and multi-scale architecture with deep supervision were implemented and trained and evaluated using the same training and test sets. The performance of each architecture was measured using the evaluation parameters defined in Section 3.3. The results, shown in Table 2, demonstrate that the proposed scheme outperforms all of the downgraded versions.

As attention UNet [29] utilizes only a 2D slice as input, it achieves the lowest performance owing to the unavailability of 3D sequential information, which plays a crucial role in distinguishing tumorous tissues from non-tumorous tissues. However, despite using 2D slice as input, due to the incorporation of attention units, attention UNet demonstrates competitive performance.

On the other hand, the 3D multi-scale network, which consists of a 3D encoder similar to the proposed SDS-MSA-Net, achieves slightly improved performance compared to the 2D attention UNet. The reason for this improvement is the incorporation of a 3D patch, consisting of five consecutive slices. The 3D sub-volume provides significantly more information than a single 2D slice, enabling the multi-scale network to achieve improved performance for brain tumor segmentation.

In the third version, we included the 2D encoder and 3D encoder with attention units similar to the proposed architecture, however, the model is trained with a conventional training mechanism. The model achieves significantly improved performance, which demonstrates the effectiveness of combining 2D and 3D inputs with attention units in a single architecture.

Incorporating a multiscale design with deep supervision in the fourth version yielded mixed results. While the model’s enhanced tumor Dice score experienced a negligible decrease, it demonstrated comparable performance for the tumor core in comparison to the prior version. Notably, whole tumor performance exhibited an improvement, which can be attributed to the use of deep supervision. This approach enables all branches of the model to focus on all outputs, namely the enhanced tumor, tumor core, and whole tumor, rather than focusing on each tumor separately, which leads to the dropped performance for enhanced tumor.

Finally, the proposed architecture with SDS training outperforms all the downgraded versions. It exhibits that the SDS framework helps the architecture to optimize the training process by selectively deeply supervising intermediate layers, subsequently, it improves the performance for the segmentation of brain tumor regions. In contrast to deep supervision, the intermediate layers are allowed to focus on one task at a time.

### 4.2. Impact of Selective Deep Supervision on Training

To train the proposed architecture effectively, a novel SDS scheme is proposed in which intermediate layers are trained to learn features pertaining to the whole and enhanced tumor regions, while only the last two deepest layers are supervised with all three tumor regions. To evaluate the effectiveness of the SDS training scheme, the proposed SDS-MSA-Net was trained with the conventional training method, in which the architecture was trained using only the output of the final layer. Additionally, the model was trained using a standard deep supervision scheme [32]. For both experiments, the models were initialized with the same random weights using a fixed seed and trained for the same number of epochs (500).

**Figure 6:**
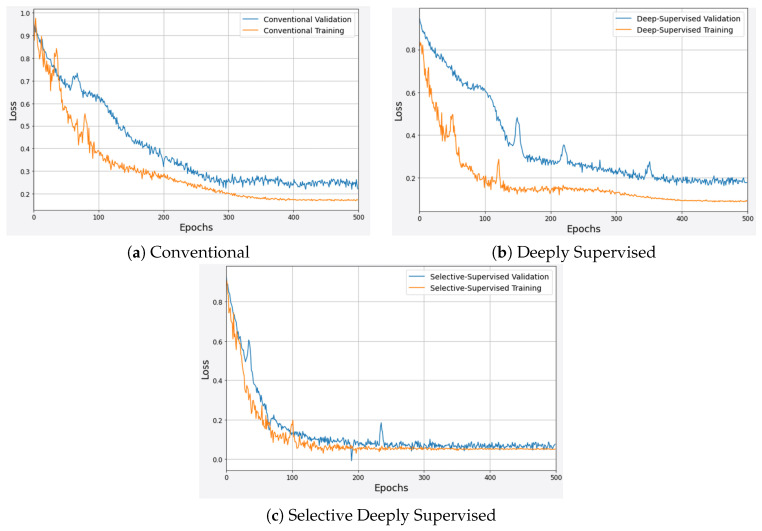
Learning curves for different training schemes.

Figure 6 shows the learning curves with conventional, standard deeply supervised, and SDS training schemes in (a), (b), and (c), respectively. The results show that the conventional method takes a long time to converge and it converges at a higher loss. On other hand, a deeply supervised network obtained fast convergence; however, there is a significant difference between training and validation loss in both models. Whereas, an SDS model not only obtains faster convergence but also, improves network optimization by achieving a lower loss. Most importantly, SDS significantly reduces the training and validation loss which enhances the generalization ability of the network.

### 4.3. Qualitative Analysis

**Figure 7:**
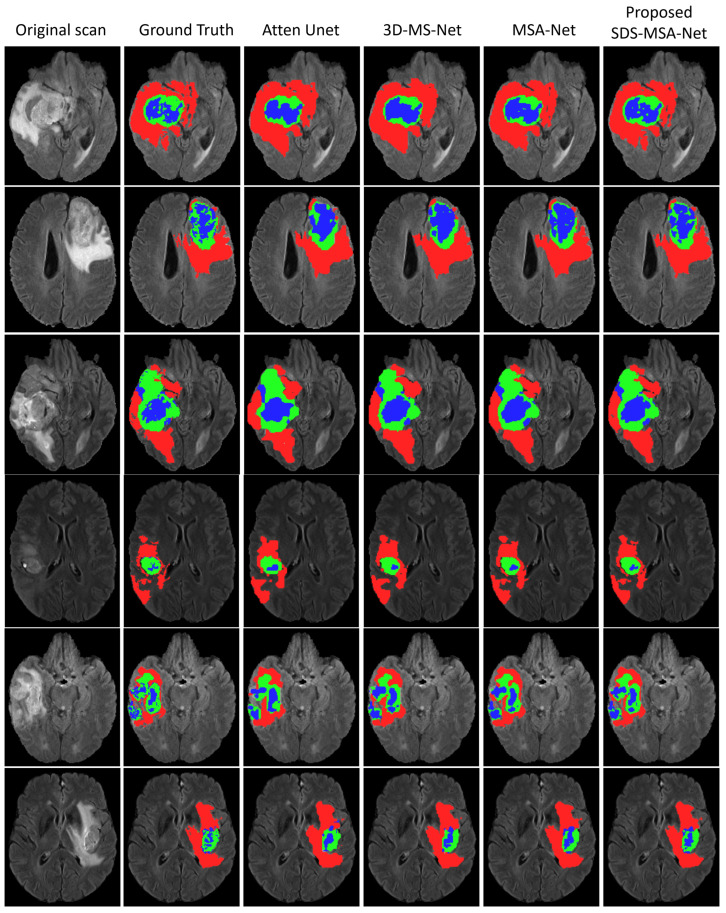
Results of SDS-MSA-Net compared with three downgraded variants (attention UNet, MS-CNN, and MSA-CNN). Note: Red, blue and green colors indicate the whole, core and enhanced tumor regions, respectively.

The proposed architecture, along with three variants of the architecture, were visually analyzed using segmentation outputs of four randomly selected samples from the test data. The results, shown in Figure 7, indicate that the model using only 2D input, known as the attention UNet, struggles with accurately segmenting all tumor regions due to the limited information available. It also demonstrates confusion between enhanced and core tumor regions. The utilization of a multi-scale network, which incorporates 3D patches, also resulted in suboptimal performance. To address these issues, the incorporation of attention units and an SDS-MSA-Net was proposed. This approach, which uses both 2D and 3D inputs in conjunction with attention units, resulted in significant improvements in segmentation performance. The proposed model showed slight enhancement in overall tumor segmentation, with particularly notable improvements in the segmentation of enhanced and core tumor regions. These findings demonstrate the effectiveness of the SDS training strategy in improving the model’s learning ability and ultimately, its performance in the segmentation of brain tumor regions.

### 4.4. Overall Performance Analysis

In order to evaluate the efficacy of the proposed method in relation to the leading techniques of BraTS2020, we implemented our model on the BraTS2020 dataset, which comprises the most extensive collection of BraTS scans currently available. The results of this comparison, as presented in Table 3, demonstrate that the proposed method demonstrates superior performance in comparison to prior state-of-the-art techniques. Notably, the proposed method exhibits a marked improvement in the segmentation of all tumor types, with particularly striking gains observed in the enhanced and core tumor segmentation. This indicates that the utilization of selective deep supervision within the proposed framework leads to the acquisition of a more informative representation, thereby improving the segmentation outcomes.

The enhancement in the overall tumor segmentation performance can be attributed to the integration of sequential information through the utilization of a five-consecutive-slice input strategy, as depicted in Figure 3, as well as the optimized multi-scale architecture that combines high-level and low-level coarse features, while incorporating attention units to effect refinement. The remarkable improvement in the segmentation of the enhanced and tumor core, however, is a result of the innovative SDS strategy, which enables the network to concentrate on the tumor region in the deeper layers, thereby facilitating the detection of the presence of enhanced tumor and tumor core regions.

## 5. Limitations

The increased inference time is a limitation of the hybrid input approach used in this study. A hybrid input approach was used, where the input was both 2D and 3D, but the output was limited to 2D. The volume of data being processed by the 3D model is much larger, which leads to a corresponding increase in computation time. The proposed approach required the inference to be taken slice by slice, and as a result, all 2D slices in a scan needed to be processed. However, with each 2D input, a 3D input is also being processed, which increases the computation time.

## 6. Conclusions

In this study, a novel approach is proposed for the segmentation and classification of brain tumors into whole, enhanced, and core regions. The proposed method for brain tumor segmentation and classification, the selective deeply supervised multi-scale attention network (SDS-MSA-Net), utilizes both 2D and 3D inputs to learn high- and low-level features related to brain tumors. Attention units are used to refine coarse features from the 2D and 3D encoding units, and the refined features are processed in a decoder block to produce the final segmentation of brain tumor regions. A novel *selective deep supervision* (SDS) scheme is also proposed, in which the intermediate layers of the decoder are selectively deeply supervised to segment different regions of the tumor based on their hierarchical structure. The proposed method was evaluated on the BraTS2020 dataset, the largest publicly available dataset for brain tumor segmentation, and was found to outperform all downgraded variants and previous state-of-the-art techniques. In our future work, we aim to explore the potential of selective deep supervision-based networks for modeling other vision- and audio-related tasks.

## Figures and Tables

**Table 1:** The aggregate quantity of MRI scans utilized for training, testing, and validation within the three BraTS datasets.

Datasets	Total No. of Scans	Total No. of Training Scans	Total No. of Validation Scans	Total No. of Testing Scans
BraTS2020	369	240	18	111
BraTS2019	335	218	17	100
BraTS2017	285	185	14	86

**Table 2:** Performances of various networks using our proposed methodology on the BraTS2020 dataset (best indicated in **bold**). Legends: whole tumor (WT), tumor core (TC), and enhancing tumor (ET).

Method	Dice			Sensitivity			Specificity			Hausdorff${}_{95}$		
	WT	TC	ET	WT	TC	ET	WT	TC	ET	WT	TC	ET
Attention-UNet [29]	86.85	71.92	72.85	84.22	68.16	76.09	83.94	79.11	80.67	7.89	10.42	9.22
3D Multi-Scale-Net	87.83	80.26	75.63	88.81	74.18	78.35	85.22	78.25	79.45	6.28	8.43	7.14
MSA-Net	88.65	84.73	78.95	89.34	87.12	81.57	88.42	81.57	84.32	5.62	7.91	5.96
DS-MSA-Net	89.19	84.96	78.81	89.87	88.59	81.94	88.02	80.93	84.10	5.11	6.91	6.04
**SDS-MSA-Net**	**90.24**	**86.93**	**80.64**	**93.16**	**90.78**	**92.41**	**92.65**	**94.12**	**93.06**	**4.27**	**6.32**	**5.87**

**Table 3:** The mean ± standard deviations of the quantitative results for various segmentation techniques on the BraTS2020 dataset is presented, with the best performance highlighted in bold. (WT: whole tumor, TC: tumor core, ET: enhancing tumor). It is worth noting that the symbol **–** signifies that the standard deviation was not reported in the corresponding study.

Method	Dice Score			Hausdorff${}_{95}$		
	WT	TC	ET	WT	TC	ET
Mina et al. [13]	90.0 ± **–**	82.0± **–**	78.0 ± **–**	5.14 ± **–**	6.64 ± **–**	7.71 ± **–**
Rupal and Mehul [2]	87.0 ± 9.3	72.0 ± 28.4	73.0 ±30.7	9.47 ± 15.2	14.53 ± 38.06	34.19 ±109.14
Laura and Veronica [18]	84.0 ± **–**	75.0 ± **–**	62.0 ± **–**	20.4 ± **–**	12.17 ± **–**	47.7 ± **–**
Hieu et al. [35]	89.99 ± **–**	84.22 ± **–**	78.43 ± **–**	5.68 ± **–**	9.56 ± **–**	24.02 ± **–**
Wang et al. [17]	90.0 ± 7.8	85.0 ± 13.0	78.0 ± 27.6	4.39 ± 7.62	8.34 ± 10.17	32.25 ± 105.54
Parvez et al. [16]	89.12 ± **–**	84.74 ± **–**	79.12 ± **–**	**–**	**–**	**–**
Lulian et al. [15]	89.85 ± **–**	82.36 ± **–**	70.07 ± **–**	13.67 ± **–**	15.62 ± **–**	99.54 ± **–**
**SDS-MSA-Net**	**90.24** ± **7.21**	**86.23** ± **10.58**	**80.64** ± **18.46**	**4.27** ± **9.56**	**6.32** ± **9.88**	**5.87** ± **22.13**

## Data Availability

All the source codes have been made available at https://github.com/Azkarehman/SDS-MSA-Net.git (accessed on 19 February 2023). This study utilized the BraTS2020 dataset which is available at https://www.med.upenn.edu/cbica/brats2020/data.html (accessed on 19 February 2023).
